# Non-Union Scoring System (NUSS): Is It Enough in Clinical Practice?

**DOI:** 10.1007/s43465-022-00767-5

**Published:** 2022-12-10

**Authors:** Diego Gaddi, Simone D. Gatti, Massimiliano Piatti, Andrea Poli, Laura De Rosa, Andrea Riganti, Giovanni Zatti, Marco Bigoni, Marco Turati

**Affiliations:** 1 Orthopedic Department, San Gerardo Hospital, University of Milano-Bicocca, Via Pergolesi 33, 20900 Monza, Italy; 2 School of Medicine and Surgery, University of Milano-Bicocca, Via Rismondo 62, Seregno, 20831 IT Monza, MB Italy; 3 Transalpine Center of Pediatric Sports Medicine and Surgery, University of Milano-Bicocca, Hospital Couple Enfant, Via Marcona 15, 20100 IT Grenoble, MI France; 4 Department of Paediatric Orthopedic Surgery, Hospital Couple Enfants, Grenoble Alpes University, Via Filzi 34, Giussano, 20833 IT Grenoble, MB France; 5 Department of Economics, Management and Quantitative Methods, University of Milano Statale, Via Speri Della Chiesa 28, 21100 IT Milan, VA Italy; 6 Department of Orthopedic Surgery, Policlinico San Pietro, Strada Statale 18, 23826 IT Mandello del Lario, LC Italy; 7 Orthopedic Department, Carate Brianza Hospital, Via Don Luigi Monza 14/B Carate B.Za, 23900 IT Lecco, LC Italy; 8 School of Medicine and Surgery, University of Milano-Bicocca, Via Pergolesi 33, 20900 Monza, Italy

**Keywords:** Non-union, Fracture, NUSS, Non-Union Scoring System, Bone defect

## Abstract

**Introduction:**

Bone consolidation defects represent a real orthopedic challenge because of the absence of validated treatment guidelines that can assist the surgeon in his choices. The aim of this study is to evaluate the appropriateness of the Non-Union Scoring System NUSS treatment protocol in the management of long bone non-unions by comparing it to the experience-based therapeutic approach carried out in our facility.

**Materials and Methods:**

We conducted a comparative outcome study of a retrospective series of 89 patients surgically treated for long bone non-union in our facility vs. clinical results reported by Calori et al. obtained following the NUSS treatment protocol.

**Results:**

Radiographic healing was reached in 13/13 non-unions (100%) in group NUSS 1, in 58/62 (93.5%) in group NUSS 2, and in 13/14 (92.9%) in group NUSS 3. The mean time to radiographic healing was 5.69 ± 2.09 months in group 1, 7.38 ± 3.81 months in group 2 and 9.23 ± 2.31 months in group 3. 91% of patients in group I, 69% in group II and 48% in group III received what would be considered by the NUSS treatment protocol an “overtreatment”, especially from a biological stand point. The comparative outcome analysis shows that our case series achieved significantly higher global healing rates (*p* value = 0.017) and shorter radiological healing times in groups NUSS 1 and 2 (*p* value < 0.001).

**Conclusion:**

From the results obtained, we can assume that the NUSS treatment protocol might underestimate the necessary therapies, particularly from a biological point of view.

## Introduction

Nowadays, long bone non-unions remain a major complication in orthopedic trauma surgery after fracture fixation and are a very complex condition to treat.

Impaired fracture healing processes represent about 5–10% of long bone fractures, even though this figure can vary considerably depending on a number of factors [1].

Despite a growing availability in treatment options, non-unions are still a condition that generates numerous discrepancies and debates among orthopedic surgeons. Accordingly, there is no general consensus regarding its classification nor treatment [2–4].

The radiological classification by Weber and Cech, defined in the 70 s, is the most frequently used classification system which divides non-unions into hypertrophic, oligotrophic and atrophic. Hypertrophic non-unions are considered to be hypervascular and with preserved biological healing potential, while atrophic pseudoarthrosis are considered to be avascular and therefore with a compromised biological status [5].

However, several studies have recently shown that there is no certain correlation between radiological characteristics and vascularity, and that atrophic non-unions are not necessarily avascular [6–8]. Over the years, other classifications have been proposed considering either bone loss, septic status or stability [9].

Although all these systems take into consideration useful elements in the clinical and therapeutic evaluation of a non-union, they consider them individually and separately, thus ignoring interactions and many other factors etiologically involved in the onset of bone consolidation failure.

With the awareness of these limits, Calori et al. developed the Non-Union Scoring System (NUSS), which represents the first multifactorial approach to non-unions taking into account 15 different items grouped in 3 macrodomains: bone condition, soft tissue status and the patients general health [10].

NUSS allows the surgeon to generate a score from 4 to 100 that correlates with the clinical complexity of a specific non-union, identifying 4 categories of severity with the relative therapeutic indications. Thanks to NUSS, we arrive at the first therapeutic protocol proposed for the management of non-unions, defined by Calori as "the Ladder Strategy", based on the so called “diamond concept” in bone regeneration [11].

According to this treatment protocol:

- Patients in class I (NUSS score 4-25) should receive a treatment aimed to improve stability, usually choosing a different system of fixation (M).  
- Patients in class II (NUSS score 26-50) require both a minor mechanical correction of fixation using the same system of fixation (m) and biological treatment with monorail therapy (b).
- Patients in class III (NUSS score 51-75) require either a major mechanical fixation associated with monorail biologic therapy (Mb) or a minor mechanical fixation associated with biologic polytherapy (mB).
- Patients in class IV (NUSS score 76-100) may require primary amputation, arthrodesis, prosthesis or mega-prosthesis implantation.

To our knowledge three studies have validated the Non-Union Scoring System as an effective classification tool and surgical therapeutic guideline [12–14]. Among these, one article also demonstrated that NUSS is a reliable system, showing substantial agreement between observers compared to the previous classification [14].

Although the Non-Union Scoring System is currently the most complete tool to classify the complexity of a non-union, in our opinion, based on our current clinical practice, it is possible that it might underestimate the extent of the necessary treatment, especially from a biological standpoint.

The aim of this study is to compare the clinical results obtained in the management of non-unions in our facility with the data in relevant literature regarding the treatment of non-unions obtained following the “ladder strategy” protocol based on the NUSS classification. Specifically, we wanted to understand if the experience-based therapeutic approach carried out in our facility differs from the NUSS protocol proposed by Calori and if there are significant differences between their relative outcomes, healing rate and radiological healing time.

The secondary endpoint was to evaluate any differences in local and systemic complication rates between the two treatments.

We decided to take as comparison in literature the article published by Calori et al.in 2014, who is among other things the creator of NUSS itself and has the biggest sample size among the articles that validated this protocol [12].

## Materials and Methods

### Study Design

This is a comparative outcome clinical study conducted in a single level-I trauma center (American college of surgeons) [15] at our institution hospital in which we retrospectively reviewed our database from November 2008 to January 2020 and selected patients surgically treated for long bone non-unions. We then compared the clinical results obtained in our facility in terms of type of treatment conducted, healing rates and radiological healing time with results reported in the article published by Calori et al. in 2014 [12].

Non-union was defined, according to the United States Food and Drug Administration (FDA) definition, as the absence of radiological signs of bone healing progression between six and nine months from the fracture time or after initial surgery in case of implant failure [16].

The inclusion criteria were as follows:

- Complete blood count performed before surgery, including ESR and CRP;
- Pharmacological history (NSAIDS and glucocorticoids);
- Minimum follow-up time of 6 months.

The exclusion criteria were the following:

- Under the age of 18;
- Multiple non-unions;
- Clavicle non-union;
- Pathological fractures;
- Immunosuppressive drug therapy and autoimmune disease;
- Psychiatric disease;
- Neoplasia and chemotherapy.

Data regarding patient characteristics (age at diagnosis and gender), the anatomical site involved and the type of initial trauma (single fracture, multiple fracture, polytrauma) were recorded. Polytrauma was defined as the presence of traumatic lesions to different organs or systems, in addition to the fracture itself, that lead to actual or potential impairment of vital parameters [17].

Written informed consent was obtained from all the patients included in this study.

The therapeutic choices that we applied were based on a clinical multi-phase evaluation of the non-union that consists of:

1.: Evaluation of systemic risk factors (smoking, drugs, diabetes, nutritional status) and any endocrinological disorders that may be present (calcium imbalances, Vit D deficiency, dysthyroidism). The objective at this stage is to correct any identified risk factors and possibly refer the patient to endocrinological visits if necessary.
2.: Detect any possible elements of local vascular impairment through a careful history of the initial trauma and subsequent surgical synthesis and possibly through more in-depth instrumental investigations such as contrast CT.
3.: Evaluation of the laxity of the non-union, of the presence of interfragmentary movements, of malalignment, of failure of synthesis or of severe osteoporosis that could cause the mobilization of screws. This was done to understand the mechanics of the non-union.
4.: Investigate infectious status: laboratory tests (CRP, neutrophilic leukocytosis), culture tests from the fistula’s serum (if present) and specific imaging such as white blood cell scan, CT scan or MRI are required if septic non-union is suspected.

When identified, septic non-unions were treated in our facility according to two different protocols.

The first protocol consists of a two-step surgical approach with debridement and implantation of Gentamicin-loaded polymethylmethacrylate PMMA cement beads or custom spacers, and subsequent removal of the cement [18].

The second protocol consists of a one-step surgery with debridement and BioActive Glass BAG S53P4 (BonAlive, BonAlive Biomaterials Ltd, Biolinja, Finland) implantation, possibly associated with other biological treatments. This is a relatively new biomaterial adopted in the later stages of this study, that has shown to have bacteriostatic, osteoconductive, osteoinductive and angiogenic properties [19–22].

In these cases, a course of post-operative antibiotic therapy was started via infectious consultation, usually of the duration of 6 weeks. Intravenous broad-spectrum antibiotic therapy (usually Meropenem and Vancomycin) was administered at first and then moved on to a specific antibiotic therapy based on the results of microbial sensitivity [23].

**Table 1:** Treatment proposed by NUSS VS Index procedure that was performed

Class	NUSS protocol indication	Index procedure	% Overtreatment
I	M	1 m 1 m + b 5 M + b 1 m + B 3 M + B	91%
II	m + b	3 m 3 M 13 m + b 8 M + b 16 m + B 19 M + B	69%
III	M + b M + B	1 m + b 7 m + B 6 M + B	43%
IV	M + B	No patients	

The overtreatment rate of the index procedure is shown in the last column

Acronyms used in this table: **M**. major mechanical stabilization using a different fixation system; **m**. minor mechanical stabilization using the same fixation system; **B**. biologic polytherapy; **b**. monorail biologic treatment

**Table 2:** Comparison between the non-union healing rates obtained with NUSS treatment protocol VS the non-union healing rates obtained in our facility after index procedure was performed

Class	Calori et al.	Healing rate	Index procedure	Healing rate	*p* value
I	60/69	86.9%	13/13	100%	0.084
II	102/117	87.1%	58/62	93.5%	0.103
III	69/84	82.1%	13/14	92.9%	0.168
Global	231/270	85.5%	84/89	94.3%	0.017

**Table 3:** Radiological time to heal

Class	Calori et al.	Index procedure	*p* value
I	8.78 ± 2.04	5.69 ± 2.09	< 0.001
II	9.02 ± 1.84	7.38 ± 3.81	< 0.001
III	9.53 ± 1.40	9.23 ± 2.31	0.329

Comparison between the radiological healing time obtained with NUSS treatment protocol VS the radiological healing time obtained in our facility

**Fig. 1:**
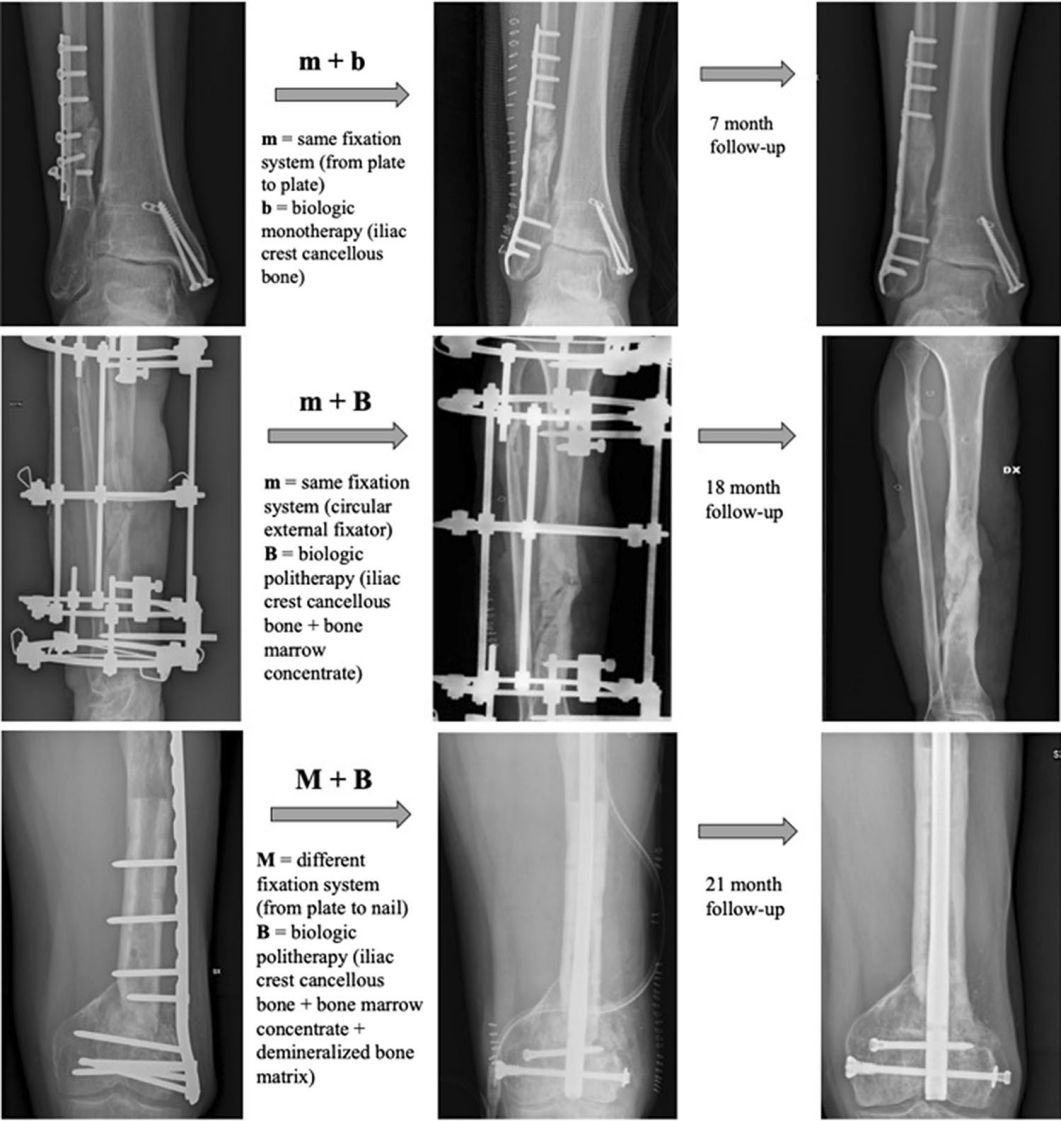
Three visual examples of how the “index procedure” was registered using the nomenclature m/M and b/B

We then registered the surgery performed to procure healing both from a mechanical (M or m) and biological (B or b) stand point, regarded as “index procedure”, and the outcomes obtained in terms of healing rates and radiological healing time. The registration of the “index procedure” was done using the same nomenclature that was used in the NUSS treatment protocol, to easily compare the two of them (visual examples are shown in Fig. 1). Intraoperative culture tests were performed systematically in all patients treated in our facility.

At last, the non-union severity index was determined using the new Calori et al.Non-Union Scoring System classification [10]. The information required to calculate the NUSS score was retrieved from electronic medical records.

### Outcome Assessments

All patients were followed up systematically at 1, 3 and 6 months after the primary treatment. Additional follow-up every 3 months was done until radiological healing (treatment success) or new diagnosis of non-union (treatment failure) occurred. Radiological success, evaluated with periodic X-rays or with CT-scan if the radiological image was not clear enough, was considered based on the presence of bridging callus in 3 out of 4 cortices (antero-posterior and latero-lateral) [12].

The radiological healing time was registered in months.

### Statistical Analysis

The sample size was calculated to assess the number needed to treat to consider as statistically significant a difference equal to four weeks (28 days in a month) and with a significance level < = 0.05 and a power 1-® = 0.95, using the standard deviation already found in the literature equal to 1.7. The minimum needed number of patients is equal to 86.9.

Within each NUSS class we compared our descriptive evidences with existing literature with respect to (i) the average time to heal and (ii) the proportion of reached healings. We use the above-mentioned tests to estimate whether and at what extent our findings are statistically different in terms of outcomes and healing time with respect to the ones obtained by Calori et al. [12]. We therefore performed T-test for the difference in proportions and for differences in mean. All statistical analysis were performed using STATA MP version 16.1 (Copyright 1985–2019 StataCorp LLC, College Station, Texas, US).

## Results

A total of 89 patients with long bone non-union were included in this clinical study. There were 65 male (73.0%) and 24 female (27.0%) patients. The mean age ± standard deviation was 43.7 ± 13.9 years (range from 18 to 77 years). Non-union was of the tibia in 33.7% of patients, the femur in 21.3%, the humerus in 20.2%, the ulna in 12.4%, the radius in 10.1% and the fibula in 4.5%. Regarding the initial trauma that led to the fracture, 46.0% of patients had a single fracture, 18.0% had multiple fractures and 36.0% had polytrauma 14 septic non-unions have been recorded (15.7%), 4 of which in class II and 10 in class III.

The NUSS score showed the following distribution: 13 patients in class I, with an average NUSS 21.2 ± 3.7; 62 patients in class II with an average NUSS 38.71 ± 7.14; 14 patients in class III with average NUSS 63.3 ± 8.1; no patients were classified in group 4.

After the index procedure we obtained the following results.

Union was achieved in 84 patients and treatment failure occurred in 5.

In class I radiographic healing was reached in 13 of 13 non-unions (100%) and the mean time to radiographic healing was 5.69 ± 2.09 months. 12 patients in class I (92.3%) underwent treatment classified higher than the proposed treatment and 1 was treated accordingly to NUSS.

In class II radiographic healing was reached in 58 of 62 non-unions (94%) and the mean time to radiographic healing was 7.38 ± 3.81 months. Of the 58 patients in class II who achieved union, 39 (67.2%) underwent treatment classified higher than the proposed treatment, 13 (22.5%) were treated accordingly and 6 (10.3%) underwent treatment classified lower than the proposed treatment. Of the 4 patients who showed treatment failure all were over treated according to the NUSS protocol.

In class III (51–75 points) radiographic healing was reached in 13 of 14 non-unions (93%) and the mean time to radiographic healing was 9.23 ± 2.31 months. Of the 13 patients in class III who achieved union, 5 (38.5%) underwent treatment classified higher than the proposed treatment, 7 (53.8%) were treated accordingly and 1 (7.7%) underwent treatment classified lower than the proposed treatment. The one patient who showed treatment failure was over treated according to the NUSS protocol. (Table 1) 

Autologous bone grafting was utilized in 58 patients (65.1%): iliac crest cancellous bone in 44 patients, bone graft using the reamer-irrigator-aspirator RIA system (Synthes, Paoli, PA, USA) in 12 patients and vascularized fibular transfer in 2 patients. In biologic polytherapy, autologous bone grafting was associated with other osteoconductive (demineralized bone matrix DBM), osteogenic (bone marrow concentrate BMC) or osteoinductive (platelet-rich plasma PRP) biological components in double or triple association. Double association was performed in 38 patients (BMC in 19 patients, PRP in 12 patients, and DBM or BAG S53P4 in 7 patients). Triple association was performed in 10 patients, in which autologous bone grafting was combined with BMC and DBM/BAG S53P4. At last, we must disclose that 4 patients underwent bone transport distraction osteogenesis, which is considered and classified as major biological treatment B.

When comparing our results with previous findings in relevant literature [12], we find that the healing rate is higher when considering both the 3 classes and the entire sample. Aggregate results are statistically significant at 5% level (*p* value = 0.017). (Table 2)

Subsequently, comparing the healing times, results show that in class I and class II the radiological healing times in our case history are significantly shorter (significance at 1% with *p *value < 0.001) compared to the data obtained from the article by Calori et al. In class I, there is an average difference of 3.09 months, while in class II there is an average difference of 1.64 months. On the contrary, in class III there is no statistical significance in the difference between the two radiological healing times (*p *value = 0.329). (Table 3)

### Septic Non-unions

A separate consideration must be made for septic non-unions. In our series, 14 patients with septic non-union with frank purulent discharge (4 in class NUSS II and 10 in class NUSS III) were identified and treated following two different surgical protocols: two-step gentamicin-load PMMA cement implantation and subsequent removal and BioActive Glass BAG S53P4 implantation.

The overtreatment rate of septic non-union was 50% (3 overtreatments in class NUSS II and 4 overtreatments in class NUSS III).

8 patients underwent the first procedure (8/14, 57.2%). Following removal of the cement, a combined biologic polytherapy B was used in all but one of these patients, in whom no biologic treatment was used. Healing was reached in all of these patients.

6 patients underwent the second procedure (6/14, 42.8%). Pure BioActive Glass BAG S53P4 was used in only one patient, while it was combined with osteogenic autologous bone graft in five patients: bone graft harvesting using the RIA in 2 patients, iliac crest cancellous bone in 2 patients and bone marrow concentrate in 1 patient 13 out of 14 of these patients achieved non-union healing with an average healing time of 8.81 ± 4.18 months.

### Complications

In our series, 4 patients out of 43 developed chronic pain at the iliac crest donor site, persistent at 3 months post operatively.

One patient developed superficial infection at the iliac crest donor site, treated with a course of systemic antibiotics and healed with no further surgical intervention.

Another patient, in which BioActive Glass was implanted, developed prolonged serous discharge with wound dehiscence, the most common and well described complication associated to BAG S53P4 [24]. This patient achieved wound healing within 2 months with no further surgical intervention.

A 6.7% (6/89) global complication rate was thus registered in our sample, slightly higher to the complication rate reported by Calori et al. of 5.5% (15/270), but with no statistical significance (*p* value 0.681) [12].

## Discussion

Non-unions remain a not fully understood complication of fracture healing in terms of classification and treatment, despite a vast number of classifications proposed and surgical options developed in last decades.

The Weber and Cech classification, which is based on X-ray imaging, although still frequently used to this day, has no univocal relation to the biological state of a non-union and therefore has a limited use in determining an adequate treatment strategy.

The Non-Union Scoring System is the first attempt to collect information about all the local and systemic etiological factors involved in non-union development and to suggest a treatment option based on the complexity of the non-union, the so called “ladder strategy”.

The main objective of this study was to compare the type of treatment performed in our department with the “ladder strategy” treatment protocol based on the Non-Union Scoring System.

The therapy performed in our facility, which we defined as “index procedure”, was based on a four-step clinical evaluation considering systemic risk factors, vascular impairment, mechanical instability and septic status.

This kind of approach allowed us to take into consideration all the etiological elements of this complex pathological condition to plan a targeted therapeutic intervention. However, although this approach is complete, doctors who do not have the same level of expertise might not be able to systematically reproduce it.

The NUSS classification allows to enclose all these etiological elements in a single score to stratify, in a standardized and comparable way, the clinical severity, and therefore the complexity of treatment, of a specific non-union.

Data from our study shows that most of our patients received what would be considered an overtreatment according to the NUSS protocol. This means that the index procedure performed was often more invasive in term of mechanical stability and/or biological support. In fact, 91% of patients in class I, 69% of patients in class II and 48% of patients in class III underwent index procedures classified higher than the treatment proposed by their NUSS score. Data also show that most of the patients who were overtreated (75.4%) received polyrail biologic treatment (B), suggesting that the nature of the higher invasiveness carried out in our facility is mainly biological.

As hypothesized by Van Basten, a more extensive treatment could result in a higher healing rate [14]. Indeed, we obtained a significantly higher global radiological healing rate, which is about 9 percentage points higher than the one reported by Calori (*p* value = 0.02).

Furthermore, our results also showed interesting differences in term of healing time. The index procedure allowed obtaining a greater number of healings with significantly shorter healing times in class I and II (*p *value < 0.001), where a more invasive treatment was frequently performed. In class I we obtained an average difference in healing times of 2.87 months, which represents a reduction of about 30% from the comparison group in literature; in class II, we obtained an average difference of 1.73 months, which represents a 20% reduction from the comparison group in literature. It is supposable that a more invasive treatment in terms of mechanical stability and/or biological support could determine higher healing rates with lower healing times in NUSS class I and II. This trend is shown in our results.

We realize that such an approach leads more quickly to a saturation of the available therapeutic options. This means that with greater treatment invasiveness, bone transports and vascularized bone transplants will be more frequently used. Unfortunately, we are aware of the limited applicability of these techniques too, which depend on the vasculo-cutaneous state of the treated segment and on the possibility of harvesting vascularized bone, leaving substitutive (prosthesis) or demolitive (amputation) surgeries as the only treatment option in the most severe cases. Furthermore, a more invasive approach can have higher direct costs and higher complication rates, therefore future studies will be necessary to assess whether the effect of such an intervention is justified by its downsides.

Another aspect worthy of further study is represented by septic non-unions. It is essential to identify and properly manage septic non-unions as they add an element of complexity to a condition that is challenging in itself [25]. In our experience two different treatment protocols were applied, each with its own variations depending on additional biologic therapies and on the mechanical fixation used, often represented by circular external fixators. Excellent non-union healing rates were obtained in this subcategory as well (13/14 patients, 92.8%), with a mean radiological healing time of 8.81 ± 4.18 months. The patient who did not heal experienced infection relapse 2 months postoperatively and underwent further surgical intervention with BAG S53P4 implantation mixed with iliac crest cancellous bone graft.

However, we must acknowledge that we only considered bone healing and radiological healing time in this paper as outcomes to define treatment success or failure. In septic non-unions however, both a fracture and a bone infection coexist, which could be classified as a diffuse osteomyelitis Cierny-Mader type IV [26]. For this reason, treatment success is not only defined by fracture healing, but also as minimum of 1 year follow-up free of infectious relapse (up to 2 years according to some authors) [27, 27]. This aspect was not integrated in our series for uniformity and simplification purposes, but it should certainly be implemented for future studies.

We recognized some limitations to this study, such as the retrospective design of the study, the absence of radiologist consultation in radiological assessment and the number of cases. Even though we conducted a retrospective study, we were able to collect all the information requested by NUSS classification. Moreover, in our institution there is limited radiologist involvement in determining treatment strategy.

At last, although the number of patients included in the study exceeded the minimum number obtained by the sample size reliability study calculation, our relatively small sample size did not allow us to ultimate some secondary considerations that we had in mind. Specifically, it would have been interesting to evaluate whether among the multitude of available treatment options it was possible to identify specific biological therapies associated with the outcome radiological healing. However, since there were few unhealed patients, any association study would have been statistically too weak.

## Conclusion

The Non-Union Scoring System NUSS was designed by Calori et al. to compare and evaluate non-unions in a more objective and systematic way. In addition to stratifying its complexity, NUSS also proposes a surgical indication for each category of severity, introducing the first treatment protocol for non-unions, called the "Ladder Strategy".

The results obtained in our study demonstrate how the “ladder strategy” treatment protocol might underestimate the therapies necessary for non-union management, particularly from a biological point of view. In fact, the patients in our study who received an “overtreatment” according to the NUSS protocol achieved a higher global healing rate with shorter healing times in class I and II. For this reason, we recognize the possibility to improve this treatment strategy with a more invasive biological approach.

It is certainly necessary to continue this line of research to propose a detailed alternative treatment protocol, which requires a much greater sample size to reach statistical significance.

## Data Availability

Marco Turati, Marco Bigoni, Massimiliano Piatti, Giovanni Zatti, Diego Gaddi, Simone Daniel Gatti, Andrea Poli, Andrea Riganti, Laura De Rosa. Drafting of the manuscript: Diego Gaddi, Simone Daniel Gatti, Marco Turati, Marco Bigoni, Giovanni Zatti, Massimiliano Piatti. Manuscript final revision: Diego Gaddi, Simone Daniel Gatti, Marco Turati, Giovanni Zatti, Marco Bigoni, Andrea Riganti. All authors approved the final version of manuscript for submission.
